# Lytic polysaccharide monooxygenases disrupt the cellulose fibers structure

**DOI:** 10.1038/srep40262

**Published:** 2017-01-10

**Authors:** Ana Villares, Céline Moreau, Chloé Bennati-Granier, Sona Garajova, Loïc Foucat, Xavier Falourd, Bodo Saake, Jean-Guy Berrin, Bernard Cathala

**Affiliations:** 1 BIA, INRA, 44300, Nantes, France; 2 Biodiversité et Biotechnologie Fongiques, INRA, Aix Marseille Univ, UMR1163, 13009, Marseille, France; 3 Chemical Wood Technology, University of Hamburg, Leuschnerstraße 91b, 21031 Hamburg, Germany

## Abstract

Lytic polysaccharide monooxygenases (LPMOs) are a class of powerful oxidative enzymes that breakdown recalcitrant polysaccharides such as cellulose. Here we investigate the action of LPMOs on cellulose fibers. After enzymatic treatment and dispersion, LPMO-treated fibers show intense fibrillation. Cellulose structure modifications visualized at different scales indicate that LPMO creates nicking points that trigger the disintegration of the cellulose fibrillar structure with rupture of chains and release of elementary nanofibrils. Investigation of LPMO action using solid-state NMR provides direct evidence of modification of accessible and inaccessible surfaces surrounding the crystalline core of the fibrils. The chains breakage likely induces modifications of the cellulose network and weakens fibers cohesion promoting their disruption. Besides the formation of new initiation sites for conventional cellulases, this work provides the first evidence of the direct oxidative action of LPMOs with the mechanical weakening of the cellulose ultrastructure. LPMOs can be viewed as promising biocatalysts for enzymatic modification or degradation of cellulose fibers.

The enzymatic deconstruction of cellulosic biomass is of great importance for global carbon cycling to face tomorrow’s environmental concerns. Cellulose is the most abundant natural biopolymer on Earth, composed of linear β-(1–4)-linked D-glucopyranose chains tightly packed, that are highly recalcitrant to enzymatic hydrolysis. This limits their cost-effective degradation and it is the main bottleneck for the development of sustainable processes including conversion of plant biomass to biofuels^1^ and production of nanocellulose-based materials^2^. Lytic polysaccharide monooxygenases (LPMOs) are considered as a breakthrough in the enzymatic degradation of cellulose^3^ because they oxidatively cleave glycosidic linkages that render the substrate more susceptible to hydrolysis by conventional cellulases. Today, LPMOs are a central component of last generation enzyme cocktails used for the industrial production of lignocellulosic ethanol^4^,^5^.

In nature, LPMOs are widespread in both bacterial and fungal kingdoms. The most abundant source of LPMOs is found in the genomes of saprotrophic fungi with some species containing more than 30 LPMO-encoding genes^6^. They are naturally secreted at an early stage of degradation when fungi are grown in the presence of cellulose or lignocellulosic material^7^,^8^. LPMOs are classified into four auxiliary activity (AA) enzyme families (AA9, AA10, AA11 and AA13) of the carbohydrate-active enzyme database (www.cazy.org)^6^. AA9 LPMOs are exclusively found in fungi and act preferentially on cellulose^9^,^10^. LPMOs breakdown cellulose by activation of oxygen, in a reducing agent-dependent manner at a copper-containing active site exposed at the surface of the enzyme^11^. Different electron donors can trigger LPMOs action including small-molecule reductants such as ascorbate, sulfur-containing species, pyrogallol or gallic acid, or more complex systems such as cellobiose dehydrogenases (CDH), glucose-methanol-choline oxidoreductases, plant-derived and fungal phenols, or photosynthetic pigments such as chlorophylls^12^,^13^,^14^,^15^. Oxidative cleavage of cellulose leads to the formation of oxidized glucose units at different positions resulting in the formation of aldonic acids at the C1 position and/or 4-ketoaldoses (gemdiols) at the C4 position.

AA9 LPMOs have a β-sandwich fold structure with a planar surface of approximately 1,200 Å^2^ to which cellulose and cello-oligosaccharides can bind^11^,^16^. The spatial position of the aromatic residues also indicates that some LPMOs could adopt a perpendicular orientation across the trajectory of the polymer suggesting binding at the surface of cellulose microfibrils^9^,^17^. So far enzymatic activity of LPMOs has been mainly evaluated through the detection of soluble oxidized products released from phosphoric acid swollen cellulose (PASC) or cello-oligosaccharides^10^,^18^,^19^,^20^,^21^,^22^,^23^,^24^,^25^,^26^,^27^,^28^. It is in this context that we report the effect of LPMOs activity on the insoluble fraction of cellulose. The characterization of remaining cellulose fibers after LPMO action reveals the structural disruption of the cellulose insoluble residue, paving the way for greater understanding of LPMO enzymatic mechanism of action.

## Results and Discussion

### LPMO induces the release of soluble oligomers from PASC but not from cellulose fibers

**Figure 1:**
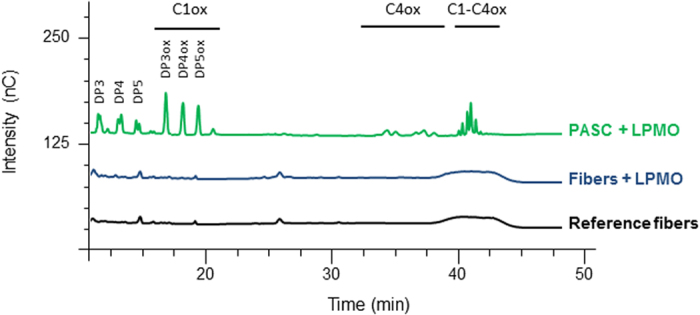
Chromatographic analysis of soluble oxidized cello-oligomers released by the action of LPMO. The chromatograms display the oligomers released from PASC treated with LPMO (green). When cellulose fibers were submitted to LPMO, no release was observed (blue). Reference (black) corresponds to cellulose fibers incubated at the same conditions but in the absence of LPMO. The chromatograms of standard oligomers (DP3–6 and DP_oxidized_3–6) are shown in Figure S1.

The LPMO enzyme from the AA9 family, *Pa*LPMO9H, originates from *Podospora anserina* and was deeply characterized in a previous study^10^. It displays activity only on polysaccharides containing (1–4)-linked glucose units (cellulose, mixed linkages beta-glucans, glucomannans and xyloglucan). However, this enzyme does not cleave plant xylans as it is the case for the AA9 LPMO from *Myceliophthora thermophila* which is so far the only AA9 LPMO able to cleave xylan only when complexed to cellulose^29^. Phosphoric acid swollen cellulose (PASC) is one of the most common substrates used to monitor cellulase activity. Preparation of PASC consists in the swelling of native cellulose in phosphoric acid so that the accessibility of cellulose chains increases. When LPMO was incubated with PASC, C1- and C4-oxidized cello-oligomers (from degree of polymerization DP3 to DP5) were released as described previously (Fig. 1)^10^. However, when LPMO was incubated with cellulose fibers, cello-oligomers were not detected in the soluble fraction (Fig. 1). This result motivated our work to identify possible modifications on cellulose fibers that are insoluble and represent a more complex and realistic substrate.

### LPMO-treated fibers are more susceptible to mechanical treatment

**Figure 2:**
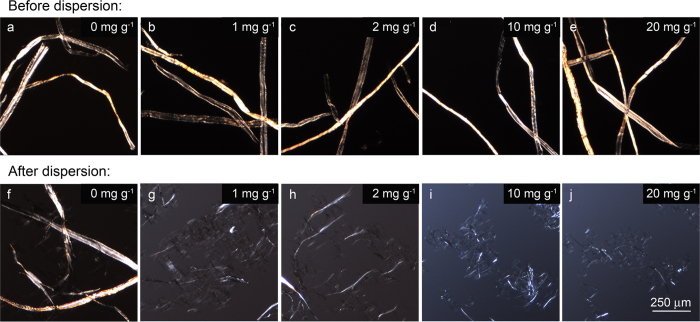
Morphology of LPMO-treated cellulose. Optical microscopy images of cellulose fibers after LPMO treatment at different enzyme concentrations (1–20 mg g^−1^) (**b–e**) before and (**g–j**) after dispersion. Images of reference samples (without LPMO treatment) have been included for non-dispersed and dispersed fibers **(a** and **f**, respectively).

The first attempt to investigate changes in the fibers structure was dedicated to the evaluation of the mechanical resistance of fibers after LPMO treatment. Original cellulose fibers are tens of micrometers in diameter and around 1 mm long, and can be visualized by optical microscopy (Fig. 2). In order to discard any effect on the fiber related to incubation conditions or to the presence of ascorbate, reference samples were subjected to the same procedure as test samples but without the addition of the LPMO enzyme. After LPMO treatment, no significant changes in physical appearance of the fibers, i.e. fibrous morphology or dimensions, were observed for all the enzyme concentrations (Fig. 2, top). This behavior was similar to endoglucanase treatments^30^,^31^, or the TEMPO oxidation^32^, which apparently do not significantly modify the fiber morphology. However, it has been demonstrated that if fibers are then subjected to chemical or mechanical treatments, significant fiber shortening and cell wall disintegration can be observed^31^,^33^,^34^,^35^. Therefore, LPMO-treated pulp material was subjected to a dispersing mechanical device followed by a short treatment by ultrasounds. Following dispersion, LPMO action resulted in fiber delamination into thinner and shorter structures (Fig. 2, bottom). The extent of surface fibrillation increased with the concentration of LPMO. As some subfibrils can be individualized at nanoscale after dispersion and cannot be detected by optical microscopy, dispersions were investigated by atomic force microscopy (AFM).

### LPMO disrupts fibers down to nanoscale

**Figure 3:**
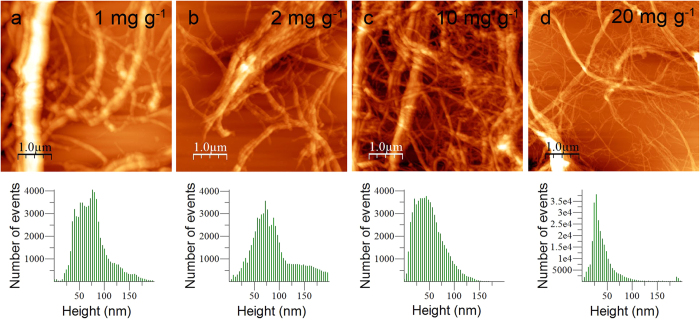
AFM imaging of LPMO-treated cellulose. (Top) AFM topography images, and (bottom) height distribution of cellulose fibers treated with LPMO at different enzyme concentrations (1–20 mg g^−1^) after dispersion.

AFM studies were carried out to get an overview of the changes in distribution of the fiber dimensions upon LPMO action. AFM micrographs of LPMO-treated samples were recorded for the different enzyme concentrations. Topography images (Fig. 3, top) show an entangled network of long fibers confirming the fibrillation at micro and nanoscale. The analysis of the images revealed different sizes of fibers for the range of LPMO concentrations studied. For the lowest enzyme concentrations (1–2 mg g^−1^), AFM images showed non-fibrillated structures together with fibers of diameters of up to 40–60 nm. As the enzyme concentration increased (10–20 mg g^−1^) better dissociation was observed and fiber dimensions reduced progressively down to elementary fibers of ca. 5 nm in diameter. The measured size corresponds to those usually reported for cellulose nanofibrils obtained by enzymatic pretreatment followed by mechanical delamination^31^, and it is in good agreement with the range of sizes measured by different methods for highly dispersed nanofibrils (tens of nm). The height distribution of the AFM images (Fig. 3, bottom) confirmed the effect of the four enzyme concentrations studied. Distributions were broad for low enzyme concentrations, which indicated the presence of large elements together with small cellulose elements. As the LPMO concentration increased, the mean height shifted towards lower values. Indeed, the mean height was around 75 nm at low enzyme concentrations (1–2 mg g^−1^) while it was 30 nm with very few large elements for the highest enzyme concentration. The decrease of fiber height clearly demonstrated that LPMO induces the disruption of fibers down to nanoscale.

### LPMO decreases the molar mass of cellulose chains

**Figure 4:**
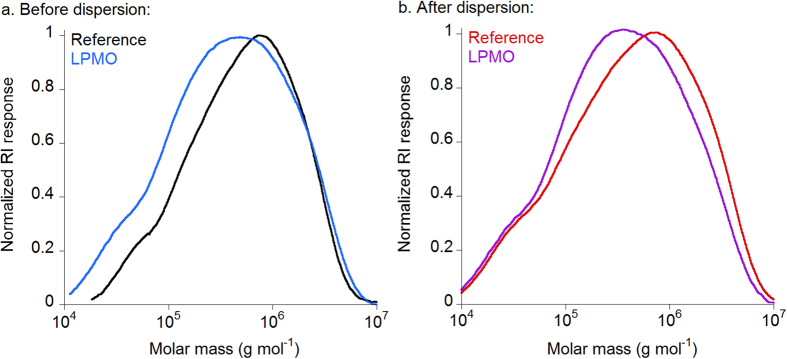
Molar mass distribution profiles of LPMO-treated cellulose. Normalized HPSEC-RI molar mass distribution profiles of reference (black and red) and LPMO-treated (blue and purple) cellulose samples (**a**) before and (**b**) after dispersion. Samples were carbanilated and eluted in N,N-dimethylacetamide containing 0.9% lithium chloride at 60 °C as described in Material and Methods.

The molar mass distribution of LPMO-treated cellulose was evaluated by high performance size exclusion chromatography coupled with multi-angle laser light scattering and refractive index detection (HPSEC-MALLS-RI) after carbanilation of samples. The highest enzyme concentration (20 mg g^−1^) was selected for SEC analysis because both AFM and optical microscopy images showed higher extent of fibrillation of pulp samples compared to other enzyme concentrations. The molar mass distribution profiles of cellulose samples treated with LPMO was characterized before and after dispersion and compared to reference samples without enzyme treatment (Fig. 4). The HPSEC-RI elution profiles are shown in Supplementary Information (Fig. S2). The weight average molar mass (*M*_*w*_), the degree of polymerization (*DP*_*w*_), and the polydispersity (*M*_*w*_*/M*_*n*_) are listed in Table 1.

Despite the broad variability in molar mass data found in literature for cellulose, the values of *M*_*w*_ and *DP*_*w*_ obtained herein (Table 1) for the starting cellulose fibers were in agreement with previous data reported^36^. The degradation of cellulose by LPMO was demonstrated by the shift towards lower *M*_*w*_and *DP*_*w*_ after the enzymatic treatment (Fig. 4, Table 1). The action of the LPMO enzyme was also demonstrated on the non-dispersed samples even if the optical microscopy images did not reveal significant changes in morphology (Fig. 2). When samples were dispersed, there was a broader distribution of molar mass, indicating the presence of high molar mass chains and the release of degradation products.

The decrease in *M*_*w*_ and *DP* after enzymatic treatment of cellulose fibers have been also observed for endoglucanase treatments. In the case of mild enzymatic pretreatments e.g. for the production of cellulose nanofibrils or for the reactivity enhancement of dissolving pulps, a broadening of the molar mass distribution and a shift in molar mass towards lower values for the endoglucanase-treated cellulose samples was observed while high molar mass fractions are preserved^30^,^37^. In these cases, the decrease in molar mass after endoglucanase treatment is mainly due to the formation of low molar mass fractions that increase polydispersity and decrease the *DP*. In the case of LPMO, although the polydispersity increased, the formation of very low molar mass fractions was not noticeable in agreement with the analysis of the soluble fraction using HPAEC.

### LPMO impacts the cellulose fiber architecture

**Figure 5:**
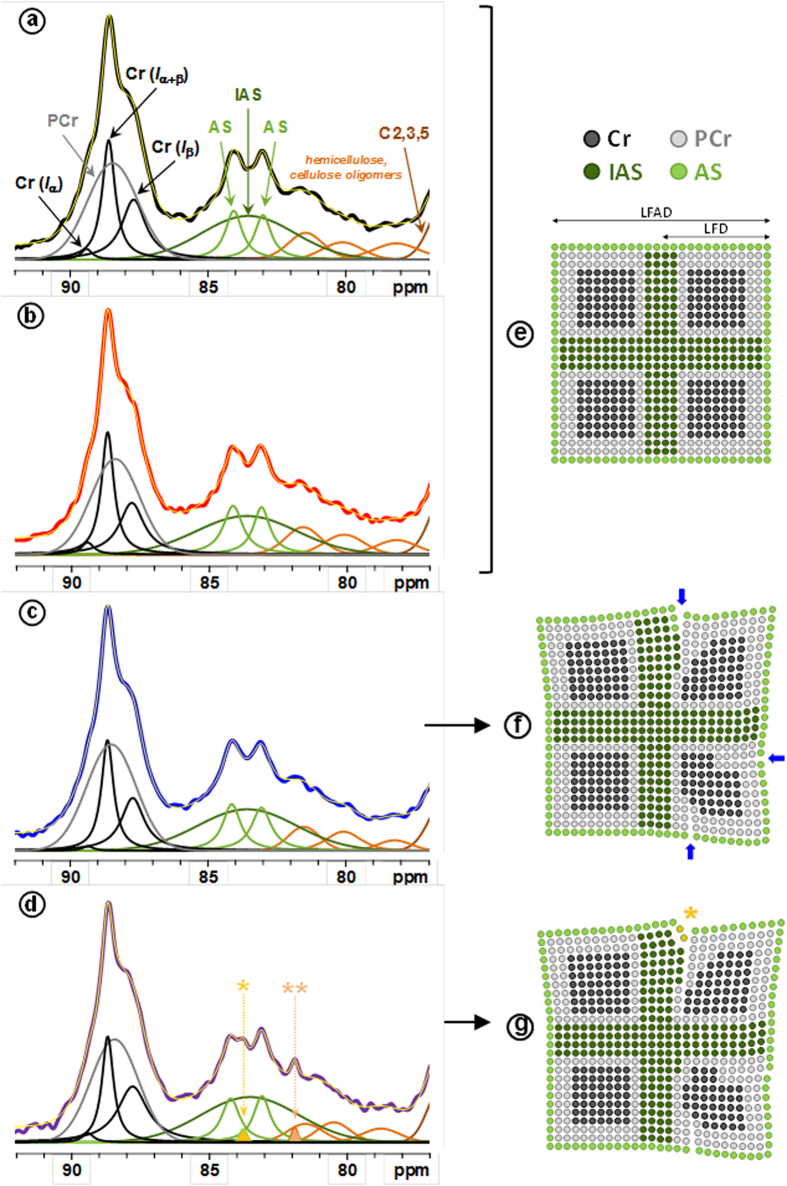
Solid-state ^13^C CP/MAS NMR spectra. Left: Deconvolution of C-4 region with crystalline forms Cr (I_α_), Cr (I_β_) and Cr (Iα_+β_) (black), para-crystalline form (PCr) (grey), accessible fibril surfaces (AS) (green), and inaccessible fibril surface (IAS) (dark green) signals for reference cellulose fibers (**a**) before and (**b**) after dispersion, and for cellulose fibers submitted to the LPMO treatment (**c**) before and (**d**) after dispersion. Four signals from hemicelluloses and/or cellulose oligomers are indicated in orange. Yellow line in (**a**,**b**,**c** and **d**) corresponds to the sum of individual peaks resulting from the spectral deconvolution. In spectrum (**d**), two additional peaks are denoted by (*) and (**) (see text for details). Right: Schematic representation of cellulose fibril model for (**e**) reference cellulose fibers, and LPMO-treated cellulose fibers (**f**) before and (**g**) after dispersion. The number of points in the scheme is proportional of the area of the corresponding NMR signals. Blue arrows represent possible nicking points created by the LPMO on the cellulose. Fibril scheme displays the different cellulose forms: lateral fibril dimension (LFD), and lateral fibril aggregate dimensions (LFAD).

To investigate deep changes induced in the fiber structure, solid-state NMR spectroscopy was used to analyze cellulose fibers before and after the LPMO action. Previous works on the action of LPMOs on PASC have demonstrated the oxidation of glucose units at C1 and C4 positions, and thus a modification of the solid-state NMR signal in the carbonyl region was expected^10^. However, the absence of the carbonyl signal in the region 180–210 ppm in the ^13^C-NMR spectra of LPMO-treated samples (Fig. S3) suggested that the carbonyl groups formed by the oxidative enzymatic action must be hydrated or linked as hemiacetals with neighboring hydroxyl groups. This behavior had been previously observed for periodate-oxidized celluloses that present a high degree of oxidation^38^,^39^. The others regions of the ^13^C CP/MAS NMR spectra show the typical distinct signals of C-1 (101–106 ppm), C-4 (78–91 ppm), C-2,3,5 carbons (68–80 ppm), and C-6 (58–68 ppm) from cellulose (Fig. S3). In an attempt to get more insights into the effects of LPMO on cellulose, ^13^C NMR spectra were deconvoluted at the C-1 and the C-4 regions (Fig. S4 and Fig. 5, respectively). Signal assignments with their corresponding values of full width at half height (FWHH) and normalized areas are compiled in Tables S1 and S2 for the C-1 and C-4 regions, respectively. At the C-1 region, no significant changes associated with the LPMO action were detected; however, an effect of mechanical treatment was observed by the broadening of the peak at 101.4 ppm from 120–130 Hz to 160 Hz for both LPMO-treated and non-treated samples. According to the assignments of this signal, it could indicate an effect of mechanical treatment on hemicelluloses.

Upon LPMO action and dispersion, deconvolution from the C-4 region revealed slight changes in signal areas at both crystalline (86–90 ppm) and amorphous (78–86 ppm) regions. Results show a slight but significant decrease of crystallinity for the LPMO-treated samples (around 56% *vs* ca. 59% for reference samples, Table 2), mostly corresponding to the increase of the inaccessible surface (IAS) area (Table S2 and Table 2). These points indicated that the LPMO induced notable changes in the cohesive zones in the elementary cellulose crystallite. In addition, the action of LPMO and dispersion resulted in the appearance of two additional signals at 83.8 and 81.9 ppm (respectively denoted by * and ** in Fig. 5d). Both signals display a narrow width (∼30 Hz) as compared to all the others signals suggesting a higher molecular mobility after LPMO action on the dispersed sample. The presence of the narrow signal at 83.8 ppm in the more disordered region of cellulose could thus be tentatively assigned to amorphous cellulosic chains accessible to the surrounding solvent after the LPMO treatment and dispersion, as illustrated in Fig. 5g by the yellow circles. The new narrow peak at 81.9 ppm could be associated with xylose in a more disorder form of the hemicellulose peak HC_*1*_ (Table S2). The sugar composition of the fibers did not reveal significant changes in the xylose content after LPMO treatment (Table S3); therefore, the new peak at 81.9 ppm can be ascribed to a higher mobility due to the combined effects of LPMO treatment and dispersion.

From the determination of crystallinity indices, the lateral fibril dimensions (LFD) can be evaluated considering a square cross-sectional cellulose microfibril model for NMR analysis^40^,^41^,^42^ (Table 2). Based on NMR signal areas from amorphous cellulose over total cellulose surfaces, and a microfibril model comprising cellulose chains having a width of 0.57 nm^43^,^44^, the LFD slightly decreased with values around 4.9 nm and 4.6 nm for reference and LPMO-treated samples, respectively, in accordance to previous published values^41^,^45^,^46^. At contrary, dimensions of the lateral fibrils aggregates (LFAD) increase from about 13 nm to 16 nm after LPMO treatment and dispersion. Both decrease of LFD and increase of LFAD may result in an increase of the fiber porosity that could favor the action of mechanical delamination and enzymatic degradation.

### Towards a LPMO mechanism of action on cellulose

Cellulose is a complex hierarchical structure where chains are arranged into elementary fibers displaying a highly ordered crystalline core inaccessible to solvents or enzymes surrounded by inaccessible less ordered chains corresponding to the contact regions between elementary fibers and rather accessible surfaces. The first evident action of LPMO is the weakening of the fiber structure upon mechanical action as demonstrated by optical microscopy and AFM studies. The increase of LPMO concentration induces intense fibrillation down to the release of individual nanofibrils whose molar mass is reduced in limited but significant manner, as the SEC analyses showed. These results indicate that the action of LPMO is likely restricted to critical nicking points of the fibers that weaken the fiber architecture (as schematically indicated by blue arrows in Fig. 5f). Solid-state NMR data indicate that this cleavage mostly occurs at the non-crystalline part of the fiber (Fig. 5e–f). Our results suggest that LPMO acts on accessible amorphous surfaces and that chains breakage and associated chemical modification induce weakening of the hydrogen bonds and van der Waals network that links adjacent chains from accessible and inaccessible surfaces. Cellulose packing is therefore disrupted and the outermost crystalline part of the fiber is affected and becomes less ordered, or para-crystalline. Upon the action of LPMO, accessible regions would be modified, which could justify the new ^13^C NMR signals detected at 83.8 and 81.9 ppm. Modification of the interaction in the inaccessible domains would also result in the decrease of the crystalline core dimensions in agreement to the slight decrease in crystallinity of the LPMO-treated cellulose and the simultaneous increase of the inaccessible surfaces proportion. Altogether, modifications reduce fiber cohesion, which induces either lower cohesion between elementary fibrils or their separation followed by their rapid association to form fibril aggregates. Both mechanisms lead to structures with higher porosity than the starting fibers. This observation is in accordance with the increase of lateral dimensions of fibril surface aggregates.

The overall effect of the LPMO on cellulose fibers can be viewed as a mechanism creating nicking points that weakens cohesion of the architecture of the fibers. This structural modification can facilitate mechanical delamination and/or creates new entry points for the attack by other hydrolytic enzymes, such as endoglucanases. In this work, we demonstrate for the first time the direct effect of LPMOs on the cellulose fiber structure. Therefore, LPMOs can be viewed not only as a biomass-degrading enzyme but also as a novel strategy for the production of nanofibers with high DP and crystallinity, and susceptible to be further transformed into novel materials.

## Materials and Methods

### Substrate

Bleached softwood kraft pulp (Stendal mill, MERCER) was used as the cellulose substrate. Cellulose fibers were hydrated in water under stirring for 48 h prior to enzymatic assays. Phosphoric acid swollen cellulose (PASC) was prepared as described previously^10^.

### Enzyme production

The lytic polysaccharide monooxygenase (LPMO) enzyme belonged to the AA9 family and originated from the coprophilic ascomycete *Podospora anserina.* The enzyme (*Pa*LPMO9H) was produced heterologously in the yeast *Pichia pastoris* and purified as described previously^10^.

### Enzymatic treatment of the pulp

The pulp (100 mg) was adjusted to pH 4.8 with acetate buffer (50 mM) in a final reaction volume of 20 mL. Purified *Pa*LPMO9H enzyme was added to the pulp at a final concentration of 1–20 mg g^−1^. L-Ascorbic acid was present at 2 mM. Enzymatic incubation was performed at 40 °C under mild agitation for 48 h. Samples were then dispersed by a Polytron PT 2100 homogenizer (Kinematica AG, Germany) for 3 min, and ultrasonicated by means of a QSonica Q700 sonicator (20 kHz, QSonica LLC., Newtown, USA) at 350 W ultrasound power for 3 min. The reference sample was submitted to the same treatment but it did not contain the LPMO enzyme. For further analysis, samples were dialyzed against ultrapure water (MWCO 12-14000) for 10 days to remove buffer, ascorbate and released soluble sugars.

### Analysis of released oxidized and non-oxidized oligosaccharides

Oxidized and non-oxidized cello-oligosaccharides generated after LPMO cleavage were analyzed by ionic chromatography (HPAEC) as previously described^47^,^48^. All assays were carried out in triplicate.

### Optical microscopy

Cellulose fibers (reference and LPMO-treated) were deposited onto a glass slide and observed by a BX51 polarizing microscope (Olympus France S.A.S.) with a 4× objective. Images (N ≥ 20) were captured by a U-CMAD3 camera (Olympus Japan).

### Atomic force microscopy (AFM)

Samples were deposited onto mica substrates from fiber solutions at 0.1 g L^−1^, and allowed to dry overnight. Topographical images on mica were registered by atomic force microscopy (AFM) by an Innova AFM (Bruker). The images were collected in tapping mode under ambient air conditions (temperature and relative humidity) using a monolithic silicon tip (TESPA, Bruker) with a spring constant of 42 N m^−1^, and a nominal frequency of 320 kHz. Image processing was performed with the WSxM 5.0 software. A series of reference images (N ≥ 6) was recorded in order to ensure the homogeneity of the sample.

### Carbanilation and size exclusion chromatography

Carbanilation was performed as previously described^49^. Briefly, water was removed from samples by freeze-drying and cellulose fibers were then dried in vacuum over phosphorous pentoxide. Samples (10 mg) were allowed to swell in anhydrous pyridine (10 mL) for 4 h in vacuum over phosphorous pentoxide. Phenylisocyanate (700 μL) was added and the reaction proceeded in a well-stirred solution for at least 48 h at 80 °C. The reaction was stopped by the addition of methanol. Pyridine was evaporated from the reaction mixture by a roto-evaporator at 40 °C and 20–40 mbar. The viscous liquid was dissolved by addition of 5 mL of acetone and agitation for 24 h. Then the acetone was evaporated in a roto-evaporator. Residual material was dried in a vacuum oven (5–10 mbar) at 40 °C under sulfuric acid and KOH for 48 h. Samples were dissolved in 10 mL of dimethylacetamide (DMAc) containing 0.9% lithium chloride (LiCl) to a concentration of 3 g L^−1^ and filtered (1, 0 μm, Rezist 30/1,0 Schleicher & Schuell, Germany) prior to size exclusion chromatography (HPSEC) analysis.

Molecular mass and degree of polymerization (DP) of carbanilated celluloses were determined by high-performance size exclusion chromatography (HPSEC-MALLS-RI). The equipment consisted of a Smartline 1050 pump (Knauer, Wissenschaftliche Geräte GmbH, Berlin, Germany), and a 1260 Infinity Quaternary autosampler (Agilent Technologies Deutschland GmbH, Germany). The HPSEC columns used were PLgel 20 μm Mixed A (3 columns 7.8 mm × 300 mm and guard column 7.8 mm × 50 mm) from Agilent, coupled in line and maintained at 60 °C using sph 99 oven (Spark Holland, Emmen, The Netherlands). The multi-detector system consisted of a MALLS instrument (Dawn Heleos, Wyatt Technology Corporation, Santa Barbara, CA, USA) and a refractive index detector (Shodex RI-101, Showa Denko Europe GmbH, Munic, Germany). Samples (100 μL) were eluted at 1.0 mL min^−1^ with DMAc containing 0.9% LiCl. Data were processed by means of the Astra^®^ software from Wyatt Technology Corporation (version 5.3.22) using 0.147 mL g^−1^ as the refractive index increment (*dn/dc*) for cellulose carbanilates. The degree of polymerization, *DP*_*w*_, was calculated by dividing the *M*_*w*_ by 519 g mol^−1^, according to the molar mass of a trisubstituted monomer of cellulose carbanilate^49^.

### Solid-state ^13^C CP/MAS NMR

Samples were firstly dialyzed (MWCO 12-14000) against ultrapure water for 10 days and secondly against dextran at 300 g L^−1^ (MWCO 3500) in order to remove water and concentrate samples for NMR measurements. Finally, samples were centrifuged at 10000 g during 30 min, and the pellets were analyzed by NMR.

Solid state ^13^C NMR experiments were performed on a Bruker Avance III 400 spectrometer operating at a ^13^C frequency of 100.62 MHz using cross-polarization magic angle sample spinning (CP/MAS). A double resonance H/X CP/MAS 4 mm probe was used. The samples were spun at a rate of 9 kHz at room temperature. The cross polarization pulse sequence parameters were: 3.7 μs proton 90° pulse, 1.75 ms contact time at 67.5 kHz, and 10 s recycle time. Typically, the accumulation of 5,120 scans was used. Spectra were referenced using the carbonyl signal of glycine at 176.03 ppm. All spectra were processed with Gaussian multiplication parameters of LB = −5 Hz and GB = 0.1.

To determine the crystallinity and the general cellulose’s morphology of the C-1 and C-4 region of the samples, we chose the sophisticated approach published by Larsson *et al*.^50^ and used several times^40^,^41^,^42^,^45^. For the C-4 region, this approach used four lines for the crystalline part and three lines for the amorphous part. In the crystalline part, three Lorentzian lines correspond to cellulose Cr (I_α_) (89.4 ppm), cellulose Cr (I_α+β_) (88.7 ppm) and cellulose Cr (I_β_) (87.8 ppm). In addition, a Gaussian line represents the para-crystalline (PCr) contribution (88.4 ppm). In the amorphous part, two Gaussian lines correspond to accessible surface (AS; 83.1 and 84.2 ppm) and one stand for inaccessible surface (IAS; 83.3–83.6 ppm). The cellulose crystallinity was determined with the peak area ratio of four lines for the crystalline part/seven lines for the cellulose C-4 region. For the C-1 region, this approach uses three Lorentzian lines, one corresponds to cellulose Cr (I_α_) (104.8 ppm) and two to cellulose Cr (I_β_) (103.7; 105.6 ppm). The Gaussian line at 104.6 ppm represents the less ordered cellulose. The pulp contained residual hemicellulose which was considered in the spectral decomposition: in the C-4 region with three lines between 77 and 82 ppm and in the C-1 region with one broad line centered at 101.5 ppm.

## Additional Information

**How to cite this article**: Villares, A. *et al*. Lytic polysaccharide monooxygenases disrupt the cellulose fibers structure. *Sci. Rep.* **7**, 40262; doi: 10.1038/srep40262 (2017).

**Publisher's note:** Springer Nature remains neutral with regard to jurisdictional claims in published maps and institutional affiliations.

## Supplementary Material

Supplementary Information

## Figures and Tables

**Table 1:** Weight average molar mass (*M*_*w*_), degree of polymerization (*DP*_*w*_), and polydispersity (*M*_*w*_ */M*_*n*_) of cellulose carbanilates produced from fibers treated with the LPMO enzyme before and after dispersion.

	Before dispersion		After dispersion	
	*Reference*	*LPMO*	*Reference*	*LPMO*
*M*_*w*_(10^3^ g mol^−1^)	895 ± 21	820 ± 14	935 ± 64	740 ± 42
*DP*_*w*_	1730 ± 40	1580 ± 30	1800 ± 120	1430 ± 80
*M*_*w*_*/M*_*n*_	4.3 ± 0.8	4.1 ± 0.1	5.6 ± 1.3	5.9 ± 0.1

Results are expressed as mean ± standard deviation (n = 3).

**Table 2:** Values of crystallinity, accessible/total fibril surface ratio (AS/(AS + IAS)), hemicellulose percentage (% HC), lateral fibril dimensions (LFD) and lateral fibril aggregate dimensions (LFAD) calculated from the C-4 region deconvolution of the solid state ^13^C CP/MAS NMR spectra of reference and LPMO-treated cellulose samples before and after dispersion.

Sample	Crystallinity	AS/(AS + IAS)	% HC	LFD (nm)	LFAD (nm)
Ref	59.2^a^ (*0.2*)	40^a^ (*2*)	13.9^a^ (*0.6*)	4.9	13.4
Ref-disp	58.5^a^ (*0.4*)	40^a^ (*1*)	13.8^a^ (*1.1*)	4.9	13.2
LPMO	56.3^b^ (*0.3*)	33^b^ (*3*)	10.2^b^ (*0.2*)	4.6	15.2
LPMO-disp	56.4^b^ (*0.2*)	33^b^ (*1*)	10.3^b^ (*0.2*)	4.6	16.2

Subscript letters indicate statistically different values (t-test, p < 0.05). Results are expressed as mean (standard deviation).
