# Supplementary material for: Lytic polysaccharide monooxygenases disrupt the cellulose fibers structure
Source: Sci Rep. 2017 Jan 10;7:40262. doi: 10.1038/srep40262 (PMC5223172; doi:10.1038/srep40262)
Supplement: Supplementary Information [file srep40262-s1.pdf]

Provided Supporting Information

Lytic polysaccharide monooxygenases disrupt the cellulose fibers structure

Ana Villares[1*], Céline Moreau[1], Chloé Bennati-Granier[2], Sona Garajova[2], Loïc Foucat[1], Xavier Falourd[1], Bodo Saake[3], Jean-Guy Berrin[2], Bernard Cathala[1]

1: BIA, INRA, 44300, Nantes, France

2: Biodiversité et Biotechnologie Fongiques, INRA, Aix Marseille Université, UMR1163, 13009, Marseille, France

3: Chemical Wood Technology, University of Hamburg, Leuschnerstraße 91b, 21031 Hamburg, Germany

Ana Villares: Ana.Villares@inra.fr

Céline Moreau: Celine.Moreau@inra.fr

Chloé Bennati-Granier: chloe.bennati-granier@ifr.ac.uk

Sona Garajova: sona.garajova@univ-amu.fr

Loïc Foucat: Loic.Foucat@inra.fr

Xavier Falourd: Xavier.Falourd@inra.fr

Bodo Saake: bodo.saake@uni-hamburg.de

Jean-Guy Berrin: jean-guy.berrin@univ-amu.fr

Bernard Cathala: Bernard.Cathala@inra.fr

I. HPAEC chromatograms of supernatants of the PACS and cellulose fibers treated with LPMO and reference chromatograms

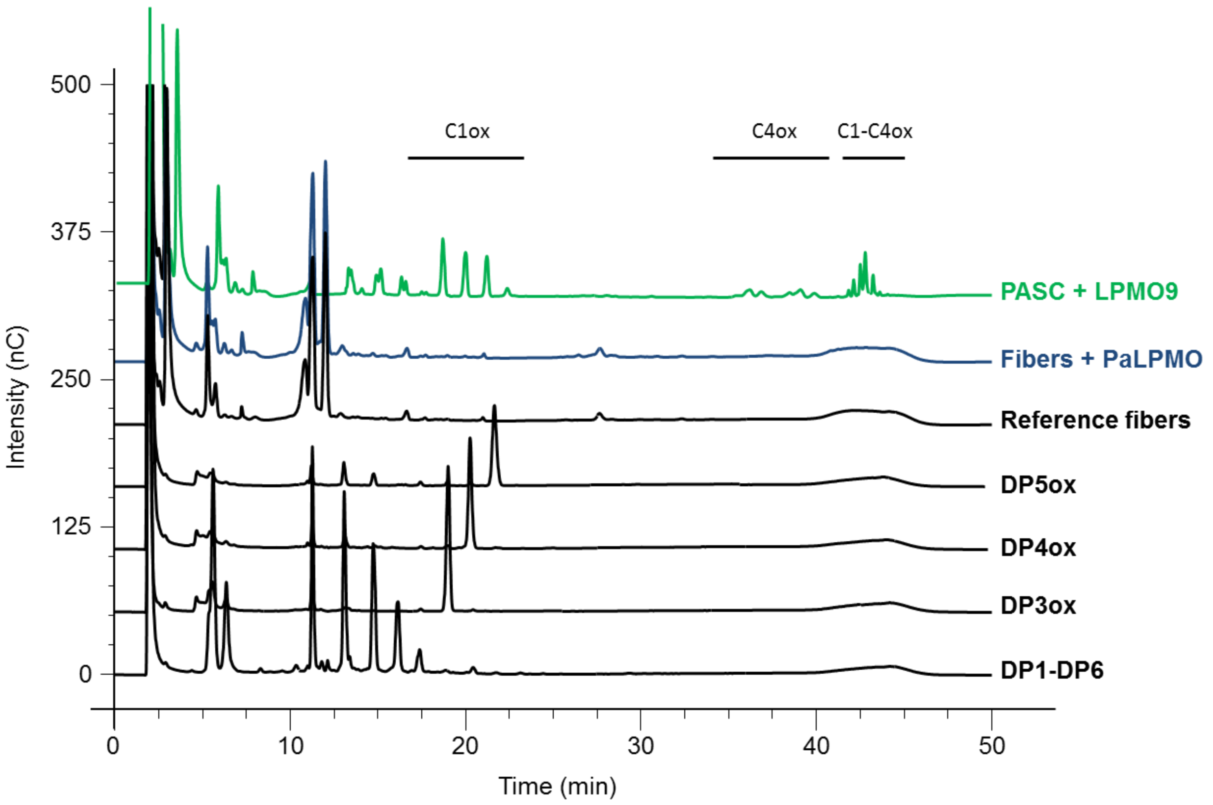

Figure S1. HPAEC analysis of soluble oxidized cello-oligomers released by the action of LPMO on cellulose. The HPAEC chromatograms display the oligomers released from PASC treated with LPMO (green); reference cellulose fibers (black); LPMO-treated cellulose fibers (blue); and reference chromatograms of standard oligomers (DP3-6 and $DP_{oxidized}$3-6).

II. SEC of cellulose fibers

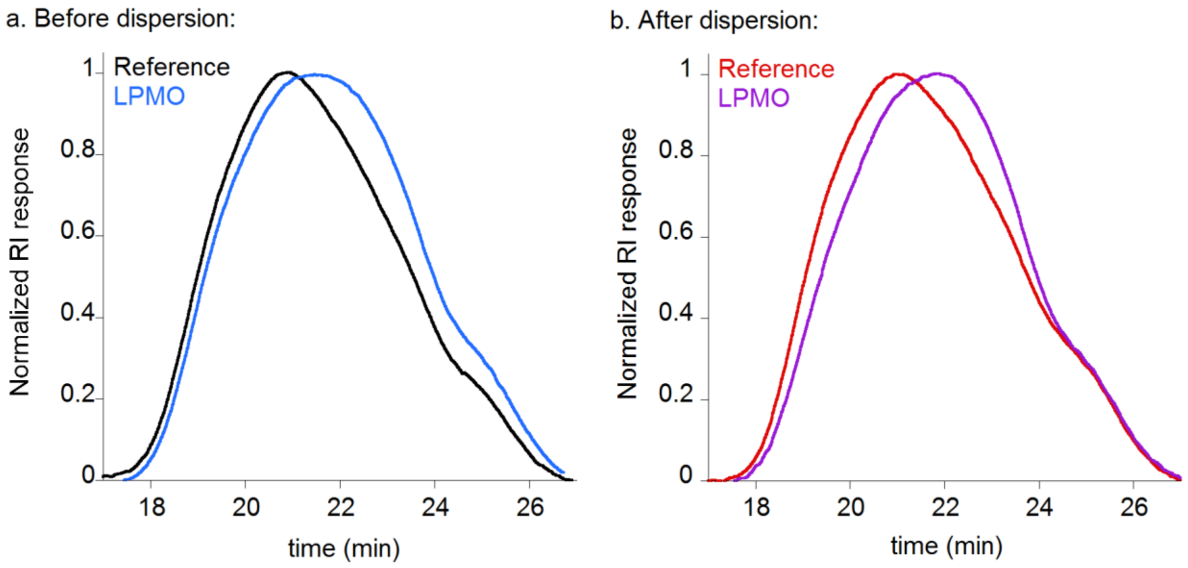

Figure S2. Normalized HPSEC-RI elution profiles of reference (black and red) and LPMO-treated cellulose (blue and purple) samples (**a**) before and (**b**) after dispersion. Samples were carbanilated and eluted in DMAc containing 0.9% LiCl at 60 ºC.

III. NMR

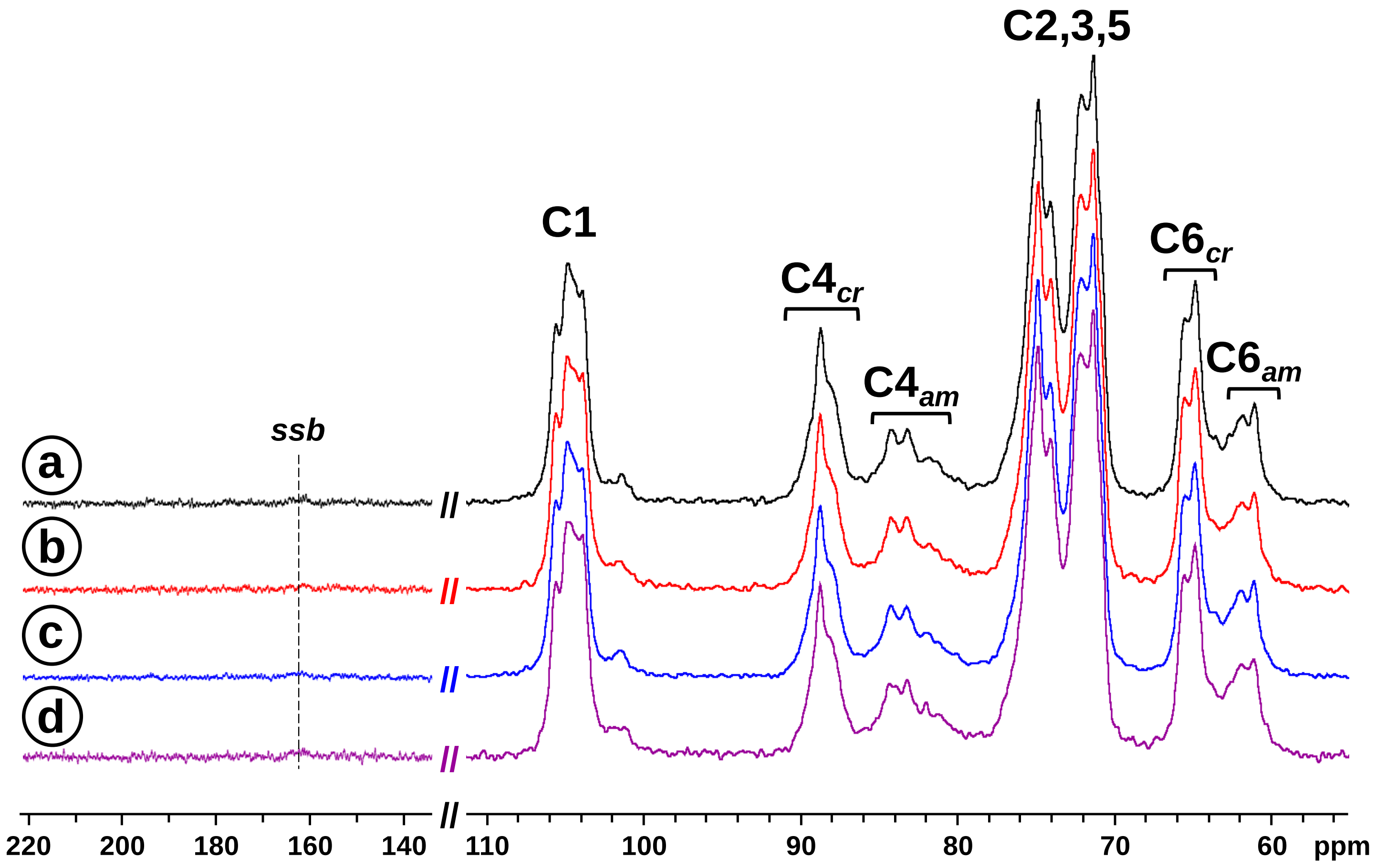

Figure S3. Solid state ^{13}C CP/MAS NMR spectra of (**a**) reference cellulose, (**b**) reference cellulose after dispersion, (**c**) LPMO-treated cellulose, and (**d**) LPMO-treated cellulose after dispersion (ssb denoted spinning side band).

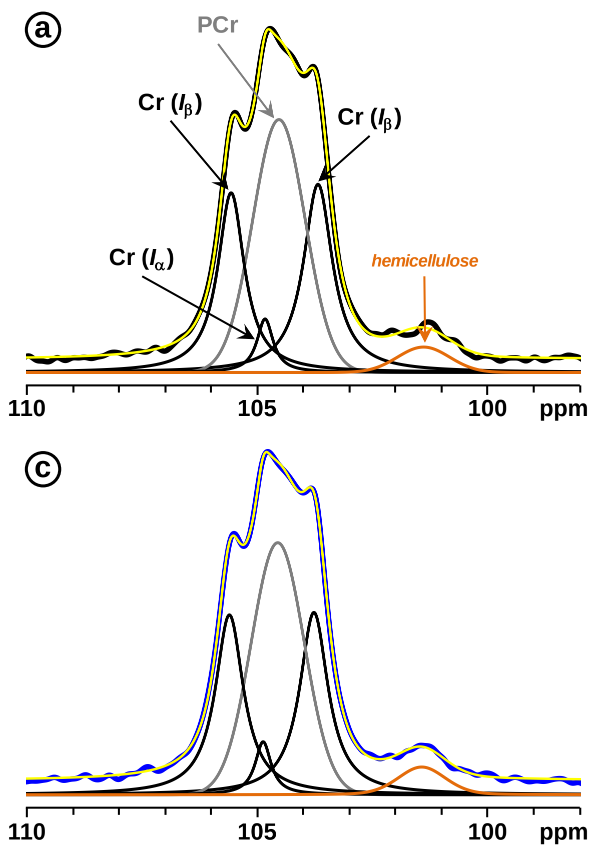

Figure S4. Deconvolution of the C-1 signals of (**a**) reference cellulose, (**b**) reference cellulose after dispersion, (**c**) LPMO-treated cellulose, and (**d**) LPMO-treated cellulose after dispersion. Cr, crystalline (black); PCr, para-crystalline (grey); and hemicellulose (orange). Yellow line in (**a**), (**b**), (**c**) and (**d**) corresponds to the sum of individual peaks resulting from the spectral deconvolution.

Table S1. Characteristics (chemical shift position of the peaks, δ; full width at half height, FWHH; and normalized area) of signals generated from the deconvolution at the C-1 region of the solid state ^{13}C CP/MAS NMR spectra of reference and LPMO-treated cellulose samples before and after dispersion. Cr, crystalline; PCr, para-crystalline; and HC, hemicellulose. Results are expressed as mean. Standard deviation (sd) from five repetition of spectral deconvolution is indicated in parenthesis. For the chemical shift δ, only sd ≥ 0.01 ppm are mentioned.

		**Peak assignment**				
		**Cr (I_β)**	**Cr (I_α)**	**PCr**	**Cr (I_β)**	**HC**
**δ (ppm)**	**Ref**	105.56	104.83	104.53 *(0.01)*	103.68	101.39
	**Ref-disp**	105.59	104.87	104.52 *(0.01)*	103.71	101.51 *(0.01)*
	**LPMO**	105.60	104.87	104.55	103.73	101.43
	**LPMO-disp**	105.59	104.87	104.53	103.72	101.48 *(0.01)*
**FWHH (Hz)**	**Ref**	71 *(1)*	44 *(3)*	132 *(2)*	77 *(1)*	132 *(2)*
	**Ref-disp**	77 *(1)*	39 *(1)*	137 *(1)*	75 *(1)*	159 *(2)*
	**LPMO**	74 *(1)*	43 *(1)*	136 *(1)*	75 *(1)*	120 *(1)*
	**LPMO-disp**	79 *(1)*	41 *(1)*	131 *(1)*	77 *(1)*	161 *(3)*
**Normalized area (%)**	**Ref**	23.5 *(0.5)*	4.5 *(0.7)*	40.8 *(1.3)*	26.9 *(0.6)*	4.2 *(0.1)*
	**Ref-disp**	26.6 *(0.1)*	4.5 *(0.1)*	40.9 *(0.3)*	23.5 *(0.2)*	4.5 *(0.3)*
	**LPMO**	24.4 *(0.1)*	4.2 *(0.1)*	42.0 *(0.1)*	24.6 *(0.1)*	4.8 *(0.1)*
	**LPMO-disp**	26.7 *(0.2)*	4.3 *(0.1)*	38.9 *(0.5)*	25.6 *(0.4)*	4.5 *(0.2)*

Table S2. Characteristics (chemical shift position of the peaks, δ; full width at half height, FWHH; and normalized area) of signals generated from the deconvolution at the C-4 region of the solid state ^{13}C CP/MAS NMR spectra of reference and LPMO-treated cellulose samples before and after dispersion. Cr, crystalline; PCr, para-crystalline; AS, accessible surfaces; IAS, inaccessible surfaces; HC_n, hemicellulose; and (*) and (**) (see text). Results are expressed as mean. Standard deviation (sd) from five repetition of spectral deconvolution is indicated in parenthesis. For the chemical shift δ, only sd ≥ 0.01 ppm are mentioned.

		**Peak assignment**							
		**Cr (I_α)**	**Cr ($I_{\alpha+\beta}$)**	**PCr**	**Cr (I_β)**	**AS**	**(*)**	**IAS**	**AS**
**δ (ppm)**	**Ref**	89.43	88.63	88.49	87.72	84.09	-	83.75 *(0.01)*	83.04
	**Ref-disp**	89.39	88.66	88.39	87.79	84.12	-	83.63 *(0.01)*	83.09 *(0.02)*
	**LPMO**	89.38 *(0.01)*	88.67	88.53	87.76	84.17	-	83.61	83.09
	**LPMO-disp**	89.37 *(0.01)*	88.66	88.42 *(0.01)*	87.75 *(0.02)*	84.21 *(0.01)*	83.76 *(0.01)*	83.51	83.08
**FWHH (Hz)**	**Ref**	50 *(1)*	55 *(1)*	212 *(1)*	102 *(1)*	75 *(2)*	-	410 *(8)*	82 *(2)*
	**Ref-disp**	56 *(1)*	54 *(2)*	209 *(1)*	109 *(1)*	82 *(2)*	-	432 *(1)*	76 *(2)*
	**LPMO**	49 *(2)*	52 *(3)*	210 *(2)*	100 *(1)*	78 *(1)*	-	423 *(1)*	82 *(2)*
	**LPMO-disp**	50 *(1)*	56 *(2)*	206 *(3)*	103 *(1)*	76 *(5)*	31 *(1)*	420 *(2)*	76 *(5)*
**Normalized area (%)**	**Ref**	1.0 *(0.1)*	12.4 *(0.2)*	26.3 *(0.1)*	11.7 *(0.1)*	6.9 *(0.3)*	-	21.7 *(0.6)*	6.1 *(0.1)*
	**Ref-disp**	1.3 *(0.1)*	12.5 *(0.4)*	26.1 *(0.9)*	10.5 *(0.2)*	7.5 *(0.3)*	-	21.6 *(0.5)*	6.7 *(0.3)*
	**LPMO**	0.9 *(0.2)*	11.5 *(0.2)*	28.0 *(0.4)*	10.3 *(0.5)*	7.0 *(0.2)*	-	26.2 *(0.7)*	5.9 *(0.3)*
	**LPMO-disp**	0.9 *(0.1)*	11.8 *(0.4)*	26.0 *(0.9)*	11.8 *(0.6)*	5.9 *(0.4)*	0.5 *(0.1)*	26.5 *(0.6)*	6.3 *(0.5)*

Table S3 (continued)

		Peak assignment			
		**(**)**	**HC_1**	**HC_2**	**HC_3**
**δ (ppm)**	**Ref**		81.49	80.16	78.20 *(0.01)*
	**Ref-disp**		81.58 *(0.01)*	80.10 *(0.01)*	78.19
	**LPMO**		81.59 *(0.02)*	80.16 *(0.02)*	78.30
	**LPMO-disp**	81.88 *(0.01)*	81.36 *(0.17)*	80.33 *(0.15)*	78.68 *(0.09)*
**FWHH (Hz)**	**Ref**		146 *(10)*	171 *(1)*	201 *(8)*
	**Ref-disp**		150 *(1)*	168 *(1)*	173 *(1)*
	**LPMO**		130 *(3)*	153 *(3)*	143 *(1)*
	**LPMO-disp**	28 *(2)*	153 *(1)*	151 *(12)*	154 *(9)*
**Normalized area (%)**	**Ref**		5.5 *(0.3)*	3.9 *(0.5)*	4.5 *(0.1)*
	**Ref-disp**		6.2 *(1.1)*	4.4 *(0.2)*	3.1 *(0.2)*
	**LPMO**		4.1 *(0.1)*	4.0 *(0.2)*	2.1 *(0.1)*
	**LPMO-disp**	0.6 *(0.1)*	3.9 *(0.3)*	3.3 *(0.3)*	2.6 *(0.3)*

Calculation of the lateral dimensions

The NMR results can be assumed as a model of $n \times n$ cellulose chains forming a crystallite with a cross-section that is roughly a square. The average number of cellulose chains, *n*, in the lateral dimension can be calculated from[1, 2]:

$$n = 2/\left(1 - \sqrt{q}\right)$$

Where q is the fraction of the total signal originating from internal cellulose chains. A conversion factor of 0.57 width ($L = 0.57n$) per cellulose chain was then used to calculate the elementary fibril and fibril aggregate dimensions. The lateral fibril dimensions (LFD) were calculated based assuming that the crystalline signals originate from the inner part of the elementary fibril, whereas both the signals from the accessible and inaccessible surfaces originate from the surface of the elementary fibril. The lateral fibril aggregate dimensions (LFAD) were calculated assuming that the surface is the accessible part and the internal signals are the inaccessible surfaces and the crystalline signals.

III. Sugar composition

Identification and quantification of neutral sugars were performed by gas-liquid chromatography (GC) after sulfuric acid degradation.[3] Sugars were reduced, acetylated and converted to alditol acetates[4].

Table S3. Chemical composition on the percent dry weight basis of reference cellulose fibers and LPMO-treated cellulose fibers. Standard deviation is indicated in parenthesis.

	Glc	Xyl	Gal	Ara	Man	Rha	Ara/Xyl
Reference	84.8	8.0	0.0	0.9	6.1	0.3	0.11
	(0.4)	(0.1)	(0.0)	(0.3)	(0.2)	(0.4)	
LPMO	83.5	7.9	0.0	1.5	6.8	0.3	0.19
	(0.3)	(0.4)	(0.0)	(0.2)	(0.1)	(0.4)	

References

1. Newman RH. Estimation of the lateral dimensions of cellulose crystallites using C-13 NMR signal strengths. *Solid State Nuclear Magnetic Resonance* **15**, 21-29 (1999).

2. Palme A, Idstrom A, Nordstierna L, Brelid H. Chemical and ultrastructural changes in cotton cellulose induced by laundering and textile use. *Cellulose* **21**, 4681-4691 (2014).

3. Hoebler C, Barry JL, David A, Delortlaval J. Rapid acid hydrolysis of plant cell wall polysaccharides and simplified quantitative determination of their neutral monosaccharides by gas-liquid chromatography. *Journal of Agricultural and Food Chemistry* **37**, 360-367 (1989).

4. Blakeney AB, Harris PJ, Henry RJ, Stone BA. A simple and rapid preparation of alditol acetates for monosaccharide analysis. *Carbohydrate Research* **113**, 291-299 (1983).
